# Recent Progress in Dendritic Cell-Based Cancer Immunotherapy

**DOI:** 10.3390/cancers13102495

**Published:** 2021-05-20

**Authors:** Kazuhiko Matsuo, Osamu Yoshie, Kosuke Kitahata, Momo Kamei, Yuta Hara, Takashi Nakayama

**Affiliations:** 1 Division of Chemotherapy, Kindai University Faculty of Pharmacy, 3-4-1 Kowakae, Higashi-osaka, Osaka 577-8502, Japan; matsuo@phar.kindai.ac.jp (K.M.); 1845110001g@kindai.ac.jp (K.K.); 2044420001m@kindai.ac.jp (M.K.); hara@phar.kindai.ac.jp (Y.H.); 2 Kindai University, 3-4-1 Kowakae, Higashi-Osaka, Osaka 577-8502, Japan; qqud7p89k@siren.ocn.ne.jp; 3 Health and Kampo Institute, 1-11-10 Murasakiyama, Sendai, Miyagi 981-3205, Japan

**Keywords:** cancer vaccine, adjuvant, dendritic cells, chemokine, XCR1, XCL1, cytotoxic T-lymphocyte

## Abstract

**Simple Summary:**

Cancer immunotherapy has now attracted much attention because of the recent success of immune checkpoint inhibitors. However, they are only beneficial in a limited fraction of patients most probably due to lack of sufficient CD8+ cytotoxic T-lymphocytes against tumor antigens in the host. In this regard, dendritic cells are useful tools to induce host immune responses against exogenous antigens. In particular, recently characterized cross-presenting dendritic cells are capable of inducing CD8+ cytotoxic T-lymphocytes against exogenous antigens such as tumor antigens and uniquely express the chemokine receptor XCR1. Here we focus on the recent progress in DC-based cancer vaccines and especially the use of the XCR1 and its ligand XCL1 axis for the targeted delivery of cancer vaccines to cross-presenting dendritic cells.

**Abstract:**

Cancer immunotherapy aims to treat cancer by enhancing cancer-specific host immune responses. Recently, cancer immunotherapy has been attracting much attention because of the successful clinical application of immune checkpoint inhibitors targeting the CTLA-4 and PD-1/PD-L1 pathways. However, although highly effective in some patients, immune checkpoint inhibitors are beneficial only in a limited fraction of patients, possibly because of the lack of enough cancer-specific immune cells, especially CD8^+^ cytotoxic T-lymphocytes (CTLs), in the host. On the other hand, studies on cancer vaccines, especially DC-based ones, have made significant progress in recent years. In particular, the identification and characterization of cross-presenting DCs have greatly advanced the strategy for the development of effective DC-based vaccines. In this review, we first summarize the surface markers and functional properties of the five major DC subsets. We then describe new approaches to induce antigen-specific CTLs by targeted delivery of antigens to cross-presenting DCs. In this context, the chemokine receptor XCR1 and its ligand XCL1, being selectively expressed by cross-presenting DCs and mainly produced by activated CD8^+^ T cells, respectively, provide highly promising molecular tools for this purpose. In the near future, CTL-inducing DC-based cancer vaccines may provide a new breakthrough in cancer immunotherapy alone or in combination with immune checkpoint inhibitors.

## 1. Introduction

Cancer is the major cause of death worldwide. Although surgery, radiation, and chemotherapy represent the three pillars of cancer therapy, the prognosis still remains poor in advanced stages of cancer often with metastases [1]. Thus, cancer immunotherapy, which aims to treat cancer by enhancing or inducing host immune responses to tumor cells, has long been hoped to become the fourth pillar of treatment. Historically, the potential therapeutic effect of host immune activity against cancer was first noted in the 19th century by Wilhelm Busch and Friedrich Fehleisen, who independently reported cancer regression after erysipelas infection [2]. Subsequently, William Coley developed Coley’s Toxin, a cocktail of killed bacteria, and used it to treat cancers such as osteosarcoma and lymphoma [2,3]. After that, Thomas and Burnet advanced the idea and proposed the cancer immunosurveillance hypothesis [2,4,5]. For decades, however, it remained difficult to induce or enhance host immune responses against tumor cells. Clinical trials such as cytokine-based immunotherapies or autologous/allogenic adoptive immune cell transfers were mostly disappointing [6]. Cancer vaccines that involve exogenous administration of tumor antigens with adjuvants to induce or enhance tumor-specific immune responses have also been tried, but mostly with unsatisfactory results [6]. Meanwhile, the concept of cancer immunosurveillance has also evolved into the theory of cancer immunoediting, which provides three phases for the complex interactions between tumors and the host immune system; namely, elimination, equilibrium, and escape [7,8]. Thus, it is now considered that, through these phases, tumor immunogenicity is edited and immunosuppressive mechanisms are acquired. However, the advent of immune checkpoint inhibitors has now revolutionized the field of cancer immunotherapy [2]. Furthermore, adoptive cell therapy employing autologous T cells with synthetic chimeric antigen receptors (CARs) has also been providing highly promising clinical results [9].

Immune checkpoint molecules negatively regulate immune responses to maintain immune homeostasis by preventing overactivated immune responses or autoimmune responses [10,11]. In this context, cancer cells often utilize the immune checkpoint pathways to suppress host antitumor immune responses [10,11]. Thus, blocking the immune checkpoint molecules was considered as a strategy to enhance host immune responses to tumor cells. Indeed, the development of checkpoint inhibitors, such as anti-CTLA4 [12], anti-PD-1 [13], and anti-PD-L1 [14], has brought remarkable success in cancer immunotherapy. However, it has also been demonstrated that not all cancer patients favorably respond to the immune checkpoint inhibitors, possibly due to insufficient CD8^+^ cytotoxic T-lymphocytes (CTLs) against tumor cells in the host [10,11]. Thus, additional strategies may be needed to elicit tumor antigen-specific CD8^+^ CTLs in patients who do not sufficiently benefit from immune checkpoint inhibitors.

It is now known that tumor cells express endogenous tumor antigens such as tumor-associated antigens (TAAs) (such as aberrantly expressed developmentally regulated antigens) and neoantigens generated by somatic mutations [15]. Therefore, if tumor antigen-specific CTLs are elicited in the host, they should be able to recognize and remove tumor cells. Thus, cancer vaccines are designed to induce tumor antigen-specific immune responses, particularly CD8^+^ CTLs [16]. Because dendritic cells (DCs) are professional antigen-presenting cells and have the unique ability to link innate and adaptive immunity, they have been regarded as the key target cells for cancer vaccine development. It is known that, upon antigen capture, DCs in peripheral tissues migrate to the draining lymph nodes where they present antigens to naive CD4^+^ and CD8^+^ T cells, resulting in the induction of effector T cells [17,18]. In the case of intracellular antigens, DCs process them and present antigen peptides through the major histocompatibility complex (MHC) class I pathway [17,18,19]. This leads to the induction of CD8^+^ CTLs. In the case of extracellular antigens, DCs usually process them and present antigen peptides through the MHC class II pathway that preferentially activates CD4^+^ T cells [18,19]. To induce CD8^+^ CTLs to extracellular antigens, therefore, the process called cross-presentation is needed to present antigen peptides via MHC class I [17]. Thus, cancer vaccines need to be preferentially processed by cross-presenting DCs. To this end, a number of adjuvants have been developed to activate cross-presenting DCs and have been shown to induce strong CD8^+^ CTL responses in animal models, yet very few have met the safety and efficacy requirement for human use [18]. Thus, it is of great importance to develop new vaccines and adjuvants that efficiently promote cross-presentation of extracellular antigens including tumor antigens for specific CD8^+^ CTL responses.

Recent research progress has shown that there are several DC subsets with different roles in the induction of antigen-specific immune responses [18,20,21]. Among the DC subsets, conventional DC1s (cDC1s) are the ones that have the ability to cross-present extracellular antigens to naive CD8^+^ T cells [22,23,24]. Thus, the success of therapeutic cancer vaccines is now considered to depend on selective and efficient antigen delivery to cross-presenting cDC1s. In this review, we first describe the phenotypic and functional characteristics of the major DC subsets and then focus on new approaches that target cross-presenting cDC1s to induce antigen-specific CD8^+^ CTLs.

## 2. DC Subsets and Their Functions

**Figure 1:**
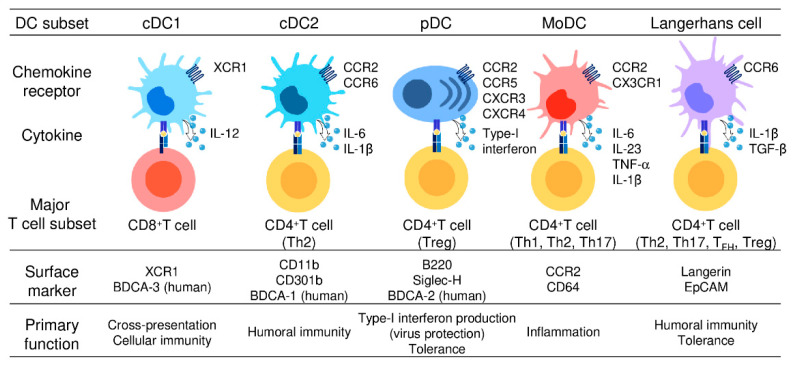
Different subsets of dendritic cells.

DCs were originally recognized for their remarkable capacity to present antigens to T cells [25,26]. Thus, DCs are regarded as the primary professional antigen-presenting cells that serve as a major link between innate and adaptive immune responses. Upon antigen capture, DCs undergo a maturation process in which they upregulate the expression of MHC molecules, costimulatory molecules (CD80/86), cytokines, chemokines, and the chemokine receptor CCR7 [27,28]. Consequently, mature DCs migrate to regional lymphoid tissues via CCR7 and activate naïve T cells to differentiate to various effector T cells, including T helper (Th) 1 cells, Th2 cells, Th17 cells, T follicular helper (T_FH_) cells, regulatory T (Treg) cells, and CD8^+^ CTLs, resulting in various T-cell responses [19,27,28]. In this context, experimental evidence accumulated over the last two decades has revealed that DCs are heterogenous and there are different DC subsets that are specialized in priming different types of effector T cells [20]. Thus, individual DC subsets are able to skew immune responses according to their induction of different effector cell responses. Almost all DC subsets in humans and mice are known to express CD11c and can be distinguished by differential expression of cell surface markers and their immunological functions [18,20,21]. DCs are now broadly divided into four major subsets; namely, conventional DCs (cDCs), plasmacytoid DCs (pDCs), monocyte-derived DCs (moDCs), and Langerhans cells (LCs). cDCs are further subdivided into cDC1s and cDC2s. Analogous subsets have been identified in humans and mice [18,20,21]. The main phenotypic and functional characteristics of these five DC subsets are described below and summarized in Figure 1.

Five DC subsets are characterized by their surface phenotypes and functional properties: cDC1s (the signature markers: XCR1 and BDCA-3), cDC2s (the signature markers: CD11b, CD301b, and BDCA-1), pDCs (the signature markers: B220, Siglec-H, and BDCA-2), moDCs (the signature markers: CCR2 and CD64), and Langerhans cells (the signature markers: Langerin and EpCAM). cDC1s are specialized for CD8^+^ CTL and Th1 cell induction and thus mediate cellular immune responses. cDC2s present antigens to both CD4^+^ T cells and CD8^+^ T cells but preferentially induce CD4^+^ Th2 induction and are involved in humoral immune responses. pDCs abundantly produce type-I interferon and are critically involved in the induction of antiviral immune responses, especially in the gut. moDCs share functional characteristics with cDC1s and cDC2s, present antigens to CD4^+^ T cells and CD8^+^ T cells, and are widely involved in inflammatory responses. LCs present antigens to both CD4^+^ T cells and CD8^+^ T cells, but preferentially promote the differentiation of naïve CD4^+^ T cells into Th2cells, T_FH_ cells, or Treg cells.

### 2.1. cDC1

Although cDC1s and cDC2s are derived from common DC precursors, they have quite different functions. cDC1s present antigens to CD8^+^ T cells via MHC class I, while cDC2s present antigens to CD4^+^ T cells via MHC class II [18,20,21]. Thus, cDC1s play a critical role in the induction of antigen-specific immune responses against intracellular pathogens and promote CD8^+^ CTL and Th1 responses [29]. Consistently, Batf3-deficient mice that lacked CD103^+^ cDC1s in the lung, intestine, mesenteric lymph nodes, skin, and skin-draining lymph nodes were shown to have reduced CD8^+^ CTL responses [30,31]. cDC1s are also known to be the most potent subset for cross-presentation of extracellular antigens to CD8^+^ T cells, although other DC subsets also have the ability for cross-presentation [32,33,34]. In mice, lymphoid tissue-resident CD8α^+^ DCs and dermal CD103^+^ DCs, which also express CD8α, were originally reported to perform cross-presentation of extracellular antigens [23,35,36]. CD141/blood dendritic cell antigen-3 (BDCA-3)^+^ DCs in humans are now considered to be the functional homolog of murine CD8α^+^ DCs [37]. Importantly, cDC1s also express dendritic and epithelial cell-205 (DEC-205), C-type lectin domain family 9 member A (CLEC9A), and XC chemokine receptor 1 (XCR1) on the cell surface in humans and mice [29,35,38,39,40]. Among the toll-like receptors (TLRs), TLR3, which recognizes double-stranded RNA, is predominantly expressed in the endosomes of cDC1s [41,42,43]. Activation of TLR3 in cDC1s leads to the abundant production of interleukin-12 (IL-12) [44], which in turn induces the production of type-II interferon (IFN-γ) by cDC1s [39,45,46]. These cytokines are involved in the induction of Th1 and CD8^+^ CTL responses. Furthermore, TLR9, which recognizes double-stranded DNA, is expressed in the endosomes of cDC1s [41]. Consistently, TLR9 agonists were shown to elicit antigen-specific CD8^+^ T-cell responses in humans [47].

### 2.2. cDC2

cDC2s are the major population of DCs in the blood and lymphoid tissues and mainly present antigens to naive CD4^+^ T cells via the MHC class II pathway. Previous studies have reported that cDC2s have the ability to induce a wide range of effector T cells, including Th1 cells, Th2 cells, Th17 cells, Treg cells, and T_FH_ cells [48,49,50,51,52]. In addition, cDC2s are also able to activate CD8^+^ T cells, although they activate CD4^+^ T cells better than CD8^+^ T cells [53]. Furthermore, cDC2s have been shown to be crucial for the induction of Th2 responses and thus play a dominant role in host defense against extracellular pathogens [48,54]. cDC2s can be identified by the surface expression of CD11b and CD301b in mice and CD1c/BDCA-1 in humans [55]. They also express several TLR family members, including TLR2, TLR4, TLR5, TLR6, TLR8, and TLR9 [41,43]. Consequently, in response to TLR stimulation, cDC2s produce various inflammatory cytokines, such as tumor necrosis factor (TNF)-α, IL-1, IL-6, IL-8, IL-12, and IL-18, as well as chemokines, such as CCL3, CCL4, and CXCL8 [43,56]. Recently, cDC2s are further subdivided into two subsets based on the expression of T-bet and RORγt [57]. Although the detailed functional characteristics of these two newly identified DC subsets are largely unknown, these subsets are reported to have different gene expression profiles and distinct metabolic and functional properties [57]. Thus, the differential expression of T-bet and RORγt in cDC2s is likely to further subdivide their immunoregulatory function.

### 2.3. pDC

pDCs were originally identified as plasmacytoid T cells or plasmacytoid monocytes on the basis of their plasma-cell morphology and the expression of lymphocyte markers or myeloid-cell markers [58]. While cDCs are mostly derived from common DC precursors, pDCs are derived not only from common DC precursors but also from common lymphoid progenitors [59,60]. Furthermore, unlike cDCs, pDCs express the lymphocyte marker B220, but not the myeloid markers CD11b and CD33 [41,55]. Recent studies have shown that pDCs are specialized for viral infection and rapidly produce large amounts of type I and III IFNs by activation via TLRs, while they have poor antigen-presenting capacity to naive T cells [41,61]. TLR7 and TLR9 are the key intracellular pattern recognition receptors, which recognize single-stranded RNA and double-stranded DNA, respectively [41,62]. pDCs play a critical role in the induction of antiviral immune responses, especially in mucosal tissues. Uncontrolled production of type-I IFN by pDCs also contributes to autoimmune diseases, including systemic lupus erythematosus and psoriasis [63,64]. Furthermore, in breast cancer and ovarian cancer, pDCs have been reported to be associated with poor prognosis by promoting expansion and activation of Treg cells via inducible costimulatory ligand (ICOSL) expression [65,66]. By using a mouse breast cancer model, it was also demonstrated that pDCs activated by TLR7 ligands exhibited antitumor activity and suppressed tumor growth in vivo [67]. In this regard, CD8α^+^ pDCs activated by TLR7 ligands were shown to exhibit direct tumor-killing activity mediated by granzyme B. Furthermore, TRAIL-expressing pDCs were shown to be capable of cross-presentation [68]. However, further studies are required to elucidate the heterogeneity and functional diversity of pDCs [69].

### 2.4. moDC

moDCs are inflammatory dendritic cells and differentiate from monocytes during infection and inflammation [70,71]. Upon infection and inflammation, monocytes robustly expand and are recruited to the inflamed sites to remove the cause of inflammation and then further differentiate into macrophages. A portion of recruited monocytes also differentiates into moDCs, which include TNF/iNOS-producing DCs (Tip-DCs) [72,73]. In mice, moDCs were originally reported to induce protective antigen-specific Th1 responses during Leishmania infection [73,74]. In humans, moDCs have been associated with several inflammatory diseases, including eczema, psoriasis, allergic rhinitis, and inflammatory bowel disease [75,76,77,78]. moDCs share many functional characteristics with cDC1s and cDC2s, such as the expression of costimulatory molecules and cytokines, as well as the ability to present antigens to CD4^+^ T cells and CD8^+^ T cells [73,79]. Accordingly, moDCs promote the differentiation of CD4^+^ T cells into Th1 cells, Th2 cells, and Th17 cells, and the differentiation of CD8^+^ T cells into CTLs [74,80,81,82,83,84]. Furthermore, moDCs are known to express CCR2 and infiltrate into inflammatory sites via CCR2, supporting the notion that they prefer to reside in inflammatory sites rather than to migrate to secondary lymph organs [85]. Taken together, moDCs are likely to be specialized in inflammatory responses. In vitro, moDCs can be easily generated from peripheral blood-derived monocytes by treating with granulocyte-macrophage colony-stimulating factor (GM-CSF) and IL-4 [79]. Taking advantage of this culture system, a wide variety of moDC vaccines has been developed and clinically tested [86]. However, moDC-based vaccines have so far shown only modest effects in the majority of cancer patients. This may be due to incomplete recapitulation of moDC development by the in vitro differentiation method. Indeed, moDCs induced by GM-CSF and IL-4 in vitro were reported to show reduced T-cell priming activity and limited migratory capacity to the lymph nodes compared with moDCs derived from peripheral blood [87,88]. Thus, further studies are needed to optimize moDC-based vaccines for clinical application.

### 2.5. LCs

LCs have long been recognized as a skin-resident DC subset with efficient migratory capacity to draining lymph nodes. However, recent studies have demonstrated that the developmental origin of LCs is from embryonic macrophage-lineage precursors, but not common DC precursors [89,90]. Furthermore, LCs can be generated from circulating monocytes under inflammatory conditions [91]. Indeed, LCs share phenotypic and functional properties with not only cDCs but also tissue-resident macrophages. LCs have also been shown to be maintained by self-renewal in the skin, as tissue-resident macrophages do [92,93]. Thus, LCs are now considered to be a unique population of tissue-resident macrophages, which are able to migrate to draining lymph nodes and present antigens to T cells. LCs can be identified by the expression of langerin/CD207, a C-type lectin, and epithelial cell adhesion molecule (EpCAM) [94,95]. Previously, many studies have demonstrated that LCs reside in the epidermal layer of the skin and serve as the first line of immunological defense [96,97]. During inflammation, activated LCs elongate their dendrites across tight junctions to capture antigens from outside [98]. Then, upon stimulation by locally produced inflammatory cytokines, such as TNF-α and IL-1β, LCs lose their connections with the surrounding epithelium and migrate to the draining lymph nodes [41]. Although LCs are able to present antigens to both CD4^+^ T cells and CD8^+^ T cells, they are dispensable for cross-presentation to induce CD8^+^ CTL responses [36]. In addition, LCs are efficient inducers of Th2, Th17, T_FH_ responses [99,100,101]. Consistently, mice lacking LCs showed impaired Th2 responses and reduced antibody production but intact Th1 responses [102,103]. Thus, when LCs sense a danger signal, they are likely to preferentially promote the differentiation of naïve CD4^+^ T cells into Th2 and T_FH_ cells, resulting in the induction of humoral immune responses. Furthermore, recent studies have shown that LCs are able to induce Treg cell differentiation [104,105]. Thus, under physiological conditions, LCs may play a role in the maintenance of immune tolerance in the skin and prevent harmful immune activation.

## 3. Use of DEC205 and CLEC9A in cDC1-Targeting Vaccines

Considerable efforts have been made to develop cancer vaccines that induce CD8^+^ CTL responses against cancer, but no sufficiently effective cancer vaccines are available so far. Because DCs were known to play a pivotal role in the induction of various immune responses, including CD8^+^ CTL responses, ex vivo-derived moDCs were also tried as DC-based cancer vaccines, but clinical outcomes were poor, possibly due to a limited capacity of in vitro-derived DCs to induce CD8^+^ CTL responses. 

Because recent studies have identified cDC1s as the key cross-presenting DC subset capable of inducing CD8^+^ CTLs [32,33,34], efforts have been focused on how to target cDC1s in antigen delivery. Because DEC-205 and CLEC9A are known to be highly expressed on cDC1s compared with other DC subsets (summarized in Table 1) [106,107,108,109,110,111,112,113,114,115,116,117,118,119,120,121,122,123], these molecules have been used as a possible target molecule to selectively deliver antigens to cDC1s [124]. DEC-205 is a C-type lectin receptor that acts as a recognition receptor for apoptotic and necrotic cells [125,126]. Although DEC-205 is expressed on all DC subsets, it is highly expressed on cDC1s and cDC2s [125,126,127,128,129]. DEC-205 is also reported to be weakly expressed on macrophages, NK cells, T cells, and B cells [125,126,127,128,129]. It was shown that coadministration of antigen-conjugated anti-DEC-205 antibodies with a combination of adjuvants such as complete Freund’s adjuvant, poly (I:C), or anti-CD40 antibody, efficiently induced antigen-specific IgG responses and CD8^+^ CTL responses [106,107,108,109]. Thus, antigen-conjugated anti-DEC-205 antibodies appeared to be capable of inducing humoral immune responses by cDC2s and cytotoxic immune responses by cDC1s. However, antigen-conjugated anti-DEC-205 antibodies alone without adjuvants failed to induce CD8^+^ CTL responses but rather induced immune tolerance [108,110]. CLEC9A is another C-type lectin receptor identified as the first receptor responsible for sensing necrotic cells [130,131,132]. CLEC9A is highly expressed on cDC1s and weakly expressed on pDCs and monocytes [40,133]. CLEC9A has also been shown to promote cross-presentation of dead cell-associated antigens [134,135]. Coadministration of antigen-conjugated anti-CLECA9 antibodies with anti-CD40 antibody efficiently induced CD8^+^ CTL responses against murine melanoma [111,112,113,114]. In the absence of adjuvants, however, antigen-conjugated anti-CLECA9 antibodies induced antigen-specific IgG responses, but not CD8^+^ CTL responses, and rather strongly induced Treg cell responses [115,116,117]. Thus, although DEC-205 and CLEC9A are promising surface molecules for targeted delivery of antigens to cDC1s, the vaccines based on these molecules require adjuvants for the efficient induction of CD8^+^ CTL responses. Furthermore, because DEC-205 and CLEC9A are also expressed on many other immune cells, their responses may be suppressive to CD8^+^ CTL responses.

## 4. Differential Expression of Chemokine Receptors by DC Subsets

Chemokines are a large family of small structurally related chemotactic cytokines that attract various leukocytes to their source of production via corresponding receptors [136,137]. Humans have around 50 chemokines, which are grouped into four subfamilies (CXC, CC, (X)C, and CX3C) by the motifs of the N-terminal conserved cysteine residues. Chemokine receptors belong to the seven-membrane G-protein-coupled receptor family, and there are 18 signal-transducing receptors in humans [136,137]. Chemokines play important roles in various biological processes, such as homeostatic migration and homing of lymphocytes, inflammatory infiltration of leukocytes, cell migration and homing during development, angiogenesis, wound healing, and even cancer metastasis [136,137,138].

Chemokine receptors are known to be differentially expressed on various DC subsets. For example, immature DCs express a wide variety of chemokine receptors such as CCR1, CCR2, CCR5, and CCR6, consistent with their ability to respond to a wide range of chemokines [139]. Upon antigen capture, however, immature DCs downregulate the expression of these chemokine receptors and upregulate CCR7, which leads them to draining lymph nodes and other secondary lymphoid tissues where its ligands CCL19 and CCL21 are constitutively produced [140,141]. Furthermore, cDC1s selectively express XCR1 [35,38]; cDC2s mainly express CCR2 and CCR6 [57,142]; pDC mainly express CCR2, CCR5, CXCR3, and CXCR4 [143,144,145]; and moDCs mainly express CCR2 and CX3CR1 [146,147]. Thus, the chemokine receptors are useful for the characterization of various DC subsets, and they may also be possible candidates for targeted delivery of antigens to specific DC subsets. Chemokines may also be used as an adjuvant to attract specific DCs at the site of vaccination. 

### 4.1. Use of Chemokines to Target DCs

Many studies were performed to examine the efficacy of chemokines as a possible vaccine adjuvant in animal models (summarized in Table 2). Some previous studies have also generated fused antigens with chemokines, such as CCL7 or CCL20, to induce antigen-specific immune responses [148,149]. Mice immunized with these chemokine fusions showed elevated antigen-specific IgG levels and protective antitumor activity against murine lymphoma [148,149]. Of note, CCL7 is a ligand for CCR1, CCR2, and CCR3, while CCL20 is a ligand for CCR6. Thus, these chemokine fusions are considered to target immature DCs. This might be the reason why these chemokine fusions successfully induce not only humoral immune responses but also antitumor CD8^+^ CTL responses, even though they do not directly target cDC1s. It was also reported that the chemokine fusions were internalized into early/late endosomal and lysosomal compartments through a clathrin-dependent process and subsequently delivered to the cytosol for proteasomal processing, facilitating cross-presentation to the MHC class I pathway [150]. However, the CCL7 receptor CCR2 is also expressed by cDC2s and pDCs, while the CCL20 receptor CCR6 by cDC2s [57,142,143,144,145]. Because cDC2s and pDCs are known to be involved in Th2 and Treg induction, respectively, activation of these DCs by the chemokine fusions might be suppressive to CD8^+^ CTL responses because of the induction of Th2 and/or Treg cells. It was further reported that fusion proteins with CCL21, a ligand for CCR7, efficiently promoted antigen-specific IgG responses, but not CD8^+^ CTL responses [149]. Of note, CCR7 is widely expressed by various activated DC subsets. Collectively, individual chemokine fusion molecules are likely to induce different responses depending on the expression pattern of the respective chemokine receptors by DC subsets. Thus, chemokine fusion vaccines might be useful to preferentially induce humoral or cellular immune responses according to the purpose. However, a chemokine fusion highly selective for cDC1 still needs to be developed for the efficient induction of CD8^+^ CTL responses.

### 4.2. The Role of the XCL1-XCR1 Axis in cDC1-CD8^+^ T-Cell Interactions

XCL1 is known to be mainly produced by CD8^+^ T cells, NK cells, and NKT cells [151,152,153]. Although XCL1 was originally reported as the first lymphocyte-specific chemokine for CD4^+^ T cells and CD8^+^ T cells and thus coined lymphotactin [151,154], such lymphocyte chemotactic activities were hardly reproduced thereafter, and its biological activity had long remained unknown even after the identification of its receptor XCR1 [155,156]. However, a breakthrough was made by the generation of XCR1 reporter mice in which lacZ was expressed under the control of the endogenous XCR1 promoter, and XCR1 was found to be exclusively expressed by CD8α^+^ cDC1s in the spleen [35]. Recombinant mouse XCL1 fused with the red fluorescent protein further confirmed that XCR1 was selectively expressed by CD103^+^ cDC1s in the skin and migratory CD103^+^ cDC1s in the draining lymph nodes [157]. Consistent with these observations, XCL1 was chemotactic for CD8^+^ cDC1s, but not for CD8^-^ cDC2s, T cells, B cells, and NK cells [35]. Furthermore, XCL1 was shown to be secreted by CD8^+^ T cells upon recognition of antigens presented by CD8^+^ cDC1s in vivo, and the deficiency of XCL1 impaired the induction of CD8^+^ CTLs by antigen-presenting CD8^+^ cDC1s [35,158]. Thus, it is now considered that the XCL1-XCR1 axis plays an important role in CD8^+^ CTL-mediated immune responses by promoting interactions of cDC1s and CD8^+^ T cells. In addition, natural killer (NK) cells are also known to secrete XCL1 and have been shown to interact with cDC1s in the tumor microenvironment [158,159]. cDC1s release cytokines such as IL-2, IL-12, and IL-18 that activate NK cells. In particular, cDC1-derived IL-12 is essential for the antitumor activity of NK cells [118,119]. In turn, activated NK cells produce cytokines such as IFN-γ, TNF-α, GM-CSF, which promote DC maturation and activation [159]. By the stimulation of such cytokines, DCs produce CXCL9, CXCL10, and CCL5 which promote the recruitment of type-I effector cells including CD8^+^ CTLs [120]. Thus, the XCL1-XCR1 axis may also play a significant role in the antitumor activity of NK cells by promoting cDC1–NK cell cross-talk in the tumor microenvironment.

### 4.3. Use of Fusion Antigens Targeting XCR1 as CTL-inducing Vaccines

**Figure 2:**
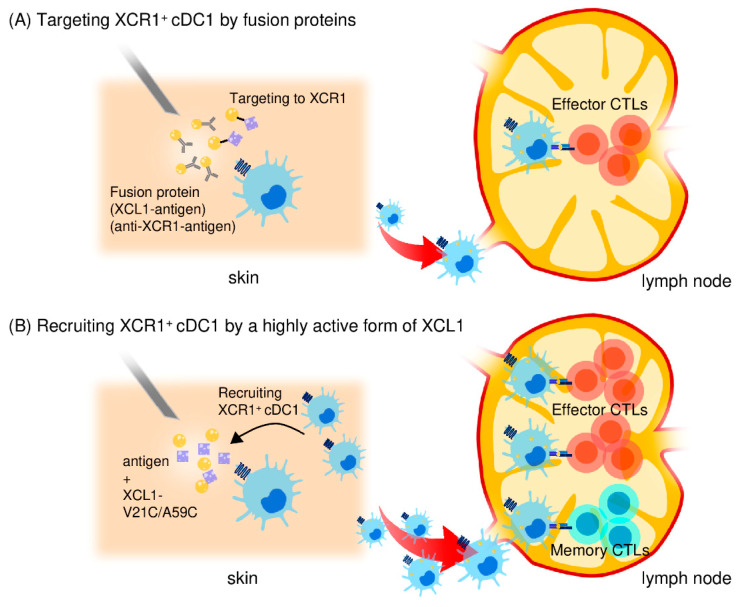
Use of the XCL1-XCR1 axis for targeted delivery of antigens to cross-presenting cDC1s. (**A**) An XCL1 protein fused to antigens or an anti-XCR1 monoclonal antibody fused to antigens can bind and deliver antigens to XCR1^+^ cDC1s to enhance antigen-specific CD8^+^ CTL responses. (**B**) A stable and highly active form of XCL1 (XCL1-V21C/A59C) efficiently induces the accumulation of cross-presenting cDC1 in the injection site. Antigen-captured cDC1s then migrate to and stay in the draining lymph nodes. Thus, by selectively attracting cDC1s, the stable and highly active XCL1 is an excellent adjuvant for induction of antigen-specific CD8^+^ CTL responses. XCL1-V21C/A59C also induces long-term memory CD8^+^ CTL responses due to prolonged maintenance of antigen-captured cDC1s in the draining lymph nodes.

As discussed above, the surface expression of XCR1 is highly selective for cDC1s in many mammalian species, including mice, sheep, macaques, and humans [121]. Thus, because of the selective expression of XCR1 on cDC1s and the critical involvement of cDC1s in CD8^+^ CTL induction, XCR1 seems to be a promising target molecule for antigen delivery to cDC1s. Thus, recent studies have utilized the XCL1-XCR1 axis to deliver antigens to cDC1s (summarized in Table 3 and Figure 2) [122,123,160,161,162]. Antigen-fused XCL1 proteins and an anti-XCR1 monoclonal antibody fused to antigens were shown to be able to bind and deliver antigens to cDC1s. Furthermore, these fusion proteins, in combination with LPS or poly (I:C) as an adjuvant, efficiently induced antigen-specific CD8^+^ CTL responses and protected mice against subsequent tumor challenge [122,123]. Similarly, dimeric XCL1-antigen fusion proteins that consisted of XCL1 and an antigen (such as ovalbumin or influenza hemagglutinin) linked through a dimerization domain consisting of the hinge and CH3 domain of human IgG3 were shown to specially bind to CD8^+^ cDC1s but not to CD11b^+^ cDC2s. The fusion proteins were also shown to efficiently induce antigen-specific CD8^+^ CTL responses and to protect mice against lethal tumor challenge or lethal influenza virus challenge [160,161]. In addition, a DNA vaccine encoding the dimeric XCL1-hemagglutinin fusion protein was also reported to induce antigen-specific CD8^+^ CTLs, resulting in full protection against lethal influenza virus challenge [162]. Taken together, the targeted antigen delivery to cDC1s via XCR1 appears to be a highly promising strategy for the induction of antigen-specific CD8^+^ CTL responses.

### 4.4. Generation of highly Active XCL1 and its Use as an Adjuvant for Antigen Delivery to cDC1s

As XCL1 is the most selective chemoattractant for cDC1s, XCL1 should be able to induce the accumulation of cDC1s in the vaccination site. Thus, XCL1 itself should be useful as a CTL vaccine adjuvant. However, XCL1 used as such failed to induce significant accumulation of XCR1^+^ cDC1s in vivo nor induce CD8^+^ CTL responses [161,163]. In this respect, XCL1 is a unique chemokine and known to have an unstable structure because of the lack of one of the two disulfide bonds that are commonly conserved in all other chemokines [151,152,153]. Accordingly, XCL1 has a relatively weak chemotactic activity. Furthermore, it has been shown that under physiological conditions, XCL1 exhibits a dynamic conformational equilibrium between two distinct structural species, the canonical chemokine form and another form that lacked XCR1 agonist activity [164]. Consistent with these observations, it has been shown that, while typical chemokines can induce cell migration with a peak response at 1–10 nM, XCL1 requires 100–200 nM to induce a peak cell migration [151,152,153,163]. Consequently, we have generated a variant form of human XCL1 termed XCL1-V21C/V59C that incorporates a second disulfide bond to stabilize the canonical chemokine structure and demonstrated that it has a highly enhanced chemotactic activity [165,166]. Thus, we hypothesized that this stable and highly active form of XCL1 used as a vaccine adjuvant would efficiently attract cDC1s and induce CD8^+^ CTL responses to coinjected antigens. Indeed, we observed that intradermal injection of XCL1-V21C/V59C with ovalbumin (OVA) as a model antigen induced the accumulation of XCR1^+^CD103^+^ cDC1s in the injection site, and OVA-loaded XCR1^+^CD103^+^ cDC1s migrated to the draining lymph nodes and stayed there for a prolonged period of time [163]. Furthermore, XCL1-V21C/V59C strongly induced OVA-specific CD8^+^ CTLs and protected mice against lethal challenge with OVA-expressing tumor cells [163]. In addition, XCL1-V21C/A59C also enhanced Th1-type cellular immune responses rather than Th2-type humoral immune responses [167]. Thus, the stable and highly active form of XCL1 provides a highly promising vaccine adjuvant for the induction of antigen-specific CD8^+^ CTLs (summarized in Table 3 and Figure 2) [163,167]. The stable and highly active form of XCL1 may also provide a better partner for fusion vaccines for targeted delivery of antigens to XCR1^+^ cDC1s.

### 4.5. Induction of Memory CD8^+^ CTLs by the Highly Active XCL1 Adjuvant

Naïve CD8^+^ T cells activated by antigen-presenting DCs differentiate not only to effector but also to memory CD8^+^ CTLs. Recent studies pointed out that the long-lasting antitumor activity of memory CD8^+^ CTLs plays a crucial role in the control of cancer [168,169]. It was also reported that long-lasting CD8^+^ CTL responses correlated well with good prognosis after cancer treatment [170]. Previously, several clinical trials have examined TLR ligands such as CpG oligodeoxynucleotide and poly (I:C) as potential adjuvants for cancer vaccines [171,172]. It is well known that DCs activated by TLR ligands produce various inflammatory cytokines, including IL-12, resulting in strong induction of Th1 and effector CD8^+^ CTL responses [173]. However, although the precise mechanism of memory CD8^+^ CTL induction has not been completely understood, recent studies have suggested that the strength of inflammation, including the production of IL-12 and IL-2, determines whether CD8^+^ T cells differentiate not only to effector but also to memory CD8^+^ CTLs [173]. Thus, strong inflammation with strong IL-12 and IL-2 signals leads to preferential induction of effector CD8^+^ CTLs, while modest inflammation with reduced IL-12 and IL-2 signals tends to induce memory CD8^+^ CTLs as well [174,175]. Moreover, the lifespan of DCs is important for the induction of memory CD8^+^ CTLs. While highly activated DCs induce strong CD8^+^ T responses, such strong CD8^+^ CTL responses rapidly eliminate antigen-carrying DCs [176,177]. Thus, strong activation of DCs by TLR ligands may possibly be disadvantageous to the induction of long-term memory CD8^+^ CTL responses. 

In this context, XCL1-V21C/A59C not only induced accumulation of XCR1^+^CD103^+^ cDC1s in the injection site and migration to draining lymph nodes but also left migratory XCR1^+^CD103^+^ cDC1s in the draining lymph nodes for a prolonged period of time compared with TLR ligands. Consistently, we observed that XCL1-V21C/A59C induced memory CD8^+^ CTL responses more efficiently than TLR ligands, as shown by the protection of mice from the challenge with OVA-expressing tumor cells after a prolonged interval [163]. Furthermore, transcutaneous administration of XCL1-V21C/V59C using a hydrophilic gel patch increased XCR1^+^CD103^+^ cDC1s in the local skin site and the draining lymph nodes for a prolonged period of time compared with the intradermal injection of XCL1-V21C/V59C, resulting in efficient induction of memory CD8^+^ CTL responses [167]. Collectively, we conclude the stable and highly active form of XCL1 has a superior quality as a CTL-inducing adjuvant because of its highly selective activity on cross-presenting cDC1s and the induction of not only effector CD8^+^ CTLs but also memory CD8^+^ CTLs (Figure 2).

## 5. Conclusions

In the last decade, much progress has been made in the development of cancer vaccines. However, current cancer vaccines still fail to induce CD8^+^ CTL responses robust enough to cure cancer. Adjuvants that strongly stimulate CD8^+^ CTL responses are still not available, and clinically approved adjuvants that primarily promote humoral immune responses are poor inducers of CD8^+^ CTL responses [178]. Thus, the development of new adjuvants to selectively stimulate CD8^+^ CTL responses is important for future success in cancer immunotherapy. In this context, TLR ligands are considered to be the most promising candidates for CTL-inducing adjuvants, but they are also known to have various adverse effects such as fever, inflammation at the injection site, and tissue damage [179,180]. Because TLR expression is found in various immune cells, including all DC subsets, T cells, B cells, and macrophages [181], the effects of TLR ligands are likely to be widespread with strong activation of various immune cells.

Many clinical trials have also been performed to investigate the effect of ex vivo activated DCs for the induction of tumor-specific CD8^+^ CTL responses in vivo [182]. Currently, sipuleucel-T, the first personalized DC vaccine for prostate cancer, has been approved by the U.S. Food and Drug Administration, but the clinical outcomes were generally poor, possibly due to the insufficient induction of CD8^+^ CTL responses. In addition, there are several clinical trials exploring the efficacy of DC-targeting cancer vaccines [183]. Most of these cancer vaccines utilize C-type lectin receptors such as DC-205 and CLEC9A for the targeted delivery of antigens to DCs including cDC1. However, these clinical trials are just beginning. On the other hand, in recent decades, there have been remarkable advances in our knowledge on the role of chemokines and chemokine receptors in antitumor immunity [184]. Interestingly, recent studies have proven that the use of some chemokines, such as CCL20, CCL21, and XCL1, as DC-targeting adjuvants is effective in eliciting humoral and cellular immune responses [185]. Among these molecules, XCL1 is a highly promising chemokine because of its selectivity to cross-presenting cDC1s. However, XCL1 is naturally a weak chemokine with an unstable structure because of the lack of one of the two C-C bonds commonly conserved in other chemokines. To circumvent this problem, we have generated a stable and highly active form of XCL1 (XCL1-V21C/A59C) by introducing a second C-C bond and demonstrated that this active form of XCL1 performs as an efficient CTL-inducing adjuvant [163,167]. On the basis of these findings, we have proposed that a stable and highly active form of XCL1 could be used as a new adjuvant selectively activating cDC1s and also as a fusion partner of cancer antigens for cDC1-targeted cancer vaccines.

Although immune checkpoint inhibitors have been approved for several cancer types and represent a major breakthrough in cancer immunotherapy, they are still largely ineffective in patients who lack sufficient anti-tumor CTLs [186,187]. Thus, a combination therapy of immune checkpoint inhibitors and cancer vaccines may be the next step to optimize cancer immunotherapy [188,189]. In fact, clinical studies of combination therapies of anti-PD-1 (nivolumab) or anti-CTLA-4 (ipilimumab) with cancer vaccines provided improved response rates in melanoma and pancreatic adenocarcinoma compared with anti-PD-1 or anti-CTLA-4 alone [190,191,192,193,194]. However, cancer vaccines that have been used in these clinical studies showed only relatively weak activity in the induction of tumor antigen-specific CD8^+^ CTL responses. Thus, cancer vaccines that efficiently target cross-presenting cDC1s would be necessary to optimize the induction of tumor antigen-specific CTLs. In this context, the stable and highly active form of XCL1 may provide a new approach to target cross-presenting cDC1s. In the near future, a combination therapy of immune checkpoint inhibitors and cancer vaccines based on molecules such as the stable and highly active form of XCL1 may greatly improve the efficacy of cancer immunotherapy.

## Figures and Tables

**Table 1:** Target molecules on cDC1s for CTL-inducing adjuvants.

Target Molecule	Type	Expressing Cell	Function	Application (Ref)
DEC-205	C-type lectin receptor	cDC1s, cDC2s, B cells, T cells, NK cells	Antigen recognition (apoptotic and necrotic cells)	[106,107,108,109,110]
CLEC9A	C-type lectin receptor	cDC1s, some pDCs, monocytes	Antigen recognition/endocytosis (cross-presentation)	[111,112,113,114,115,116,117]
XCR1	Chemokine receptor	cDC1s	Cell migration	[118,119,120,121,122,123]

**Table 2:** Use of chemokines as an adjuvant.

Chemokine	Chemokine Receptor	Immune Response	Ref.
CCL7	CCR1, CCR2, CCR3	Antigen-specific IgG response Antitumor effect	[149,150]
CCL20	CCR6	Antigen-specific IgG response Antitumor effect	[149,150]
CCL21	CCR7	Antigen-specific IgG response	[150]
XCL1	XCR1	CTL response, Antitumor effect Antiviral effect	[118,119,120,121,122,123]

**Table 3:** Vaccination strategy employing the XCR1-XCL1 axis for the induction of CD8^+^ CTLs.

Type	Immunization Method	Immune Response	Ref
Targeting cDC1			
Antigen-XCL1 fusion protein	Injection	CTL responses combined with LPS	[118]
Antigen-conjugated anti-XCR1 antibody	Injection	CTL responses combined with LPS	[118]
Antigen peptide-XCL1 fusion protein	Injection	CTL responses, antitumor immunity, enhance CTL responses with anti-PD1	[119]
Antigen-XCL1 dimeric fusion protein	DNA vaccine	CTL responses, Influenza virus protection	[120,122]
	Laser-assisted intradermal delivery	CD4+/CD8+ T-cell responses, antitumor immunity	[121]
Recruiting cDC1			
Mixture of antigen and a highly active form of XCL1	Injection	Effector and memory CTL responses, antitumor immunity	[123]
	Transcutaneous device delivery	Enhance memory CTL responses, antitumor immunity	[163]

## Data Availability

Not applicable.
